# Inhibitory effect of lignin on the hydrolysis of xylan by thermophilic and thermolabile GH11 xylanases

**DOI:** 10.1186/s13068-022-02148-4

**Published:** 2022-05-14

**Authors:** Miriam Kellock, Jenni Rahikainen, Anna S. Borisova, Sanni Voutilainen, Anu Koivula, Kristiina Kruus, Kaisa Marjamaa

**Affiliations:** 1 VTT Technical Research Centre of Finland Ltd, P.O. Box 1000, 02044 VTT Espoo, Finland; 2 Aalto University, P.O. Box 16100, 00076 Aalto Espoo, Finland

**Keywords:** Thermal stability, Adsorption, Binding, Inhibition, Glycoside hydrolase, Xylanase

## Abstract

**Background:**

Enzymatic hydrolysis of lignocellulosic biomass into platform sugars can be enhanced by the addition of accessory enzymes, such as xylanases. Lignin from steam pretreated biomasses is known to inhibit enzymes by non-productively binding enzymes and limiting access to cellulose. The effect of enzymatically isolated lignin on the hydrolysis of xylan by four glycoside hydrolase (GH) family 11 xylanases was studied. Two xylanases from the mesophilic *Trichoderma reesei*, TrXyn1, TrXyn2, and two forms of a thermostable metagenomic xylanase Xyl40 were compared.

**Results:**

Lignin isolated from steam pretreated spruce decreased the hydrolysis yields of xylan for all the xylanases at 40 and 50 °C. At elevated hydrolysis temperature of 50 °C, the least thermostable xylanase TrXyn1 was most inhibited by lignin and the most thermostable xylanase, the catalytic domain (CD) of Xyl40, was least inhibited by lignin. Enzyme activity and binding to lignin were studied after incubation of the xylanases with lignin for up to 24 h at 40 °C. All the studied xylanases bound to lignin, but the thermostable xylanases retained 22–39% of activity on the lignin surface for 24 h, whereas the mesophilic *T. reesei* xylanases become inactive. Removing of N-glycans from the catalytic domain of Xyl40 increased lignin inhibition in hydrolysis of xylan when compared to the glycosylated form. By comparing the 3D structures of these xylanases, features contributing to the increased thermal stability of Xyl40 were identified.

**Conclusions:**

High thermal stability of xylanases Xyl40 and Xyl40-CD enabled the enzymes to remain partially active on the lignin surface. N-glycosylation of the catalytic domain of Xyl40 increased the lignin tolerance of the enzyme. Thermostability of Xyl40 was most likely contributed by a disulphide bond and salt bridge in the N-terminal and α-helix regions.

**Graphical Abstract:**

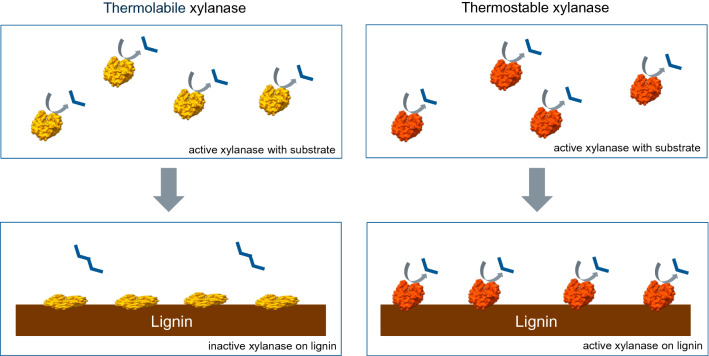

**Supplementary Information:**

The online version contains supplementary material available at 10.1186/s13068-022-02148-4.

## Background

Biotechnical conversion of lignocellulose to fuels, chemicals and other valuable products is a core technology in transformation to bioeconomy. Conversion of lignocellulosic biomass to sugars includes physico-chemical pretreatment to disrupt the cell wall structure, enzymatic hydrolysis of the polysaccharides, and microbial conversion of the released sugars into desired products. The cost of enzymes is still one of the big challenges in large-scale utilisation of lignocelluloses and the efficiency of enzymes could be improved [1].

Endo-1,4-β-xylanases (EC 3.2.1.8) are important industrial enzymes used in the lignocellulose biorefineries, in bleaching of pulp, and in food and feed applications [2]. In lignocellulose saccharification even a small quantity of xylan in the pretreated biomass can restrict the complete hydrolysis [3]. This can be mitigated by supplementing lignocellulolytic enzyme cocktails with a sufficient amount xylanase activity [4]. Consequently, xylanases are included in the commercial enzyme products designed for lignocellulose saccharification and preservation of xylanase activity throughout the hydrolysis is essential for the hydrolysis efficiency.

The commonly used hydrothermal pretreatments dissolve hemicelluloses as well as partially dissolve, but mostly redistribute lignin, thus improving enzyme access to cellulose [5]. High temperature and acidic conditions applied in the lignocellulose pretreatment also change the lignin structure making the lignin more surface active towards proteins, consequently causing non-productive binding and inactivation of the hydrolytic enzymes [6–8]. Biochemical and structural properties of enzymes, such as high surface hydrophobicity, positive surface charge and the presence of a carbohydrate-binding module (CBM) from family CBM1, are known to increase binding to lignin [7, 9–11]. Cellulases and accessory enzymes can also be inhibited by soluble lignin-derived compounds [12]. Interestingly, it has been shown that in the case of cellobiohydrolases, the key enzymes in hydrolysis of crystalline cellulose, high thermal stability protects the enzyme from inactivation on the lignin surface [13]. Effect of lignin on xylanases is much less studied but both hydrophobic interactions [14] and electrostatic interactions [11, 15] have been proposed to be involved in binding of xylanases onto lignin. On the other hand, some xylanases have been reported to be activated by soluble lignin-derived phenolics or monophenols [16, 17].

In the Carbohydrate Active Enzyme database (CAZy, http://www.cazy.org/), endo-1,4-β-xylanase (EC 3.2.1.8) activity is found in seven glycoside hydrolase (GH) families. Most xylanases are categorised into GH families 10 and 11. GH11 xylanases have higher substrate specificity and hydrolyse longer xylo-oligomers than GH10 xylanases [18]. *Trichoderma reesei*, the well-known filamentous fungus employed in industrial protein production, produces two major GH11 xylanases TrXyn1 and TrXyn2 [19]. Both are single-domain enzymes. Major differences between these enzymes are the isoelectric point (i.e. surface charge), thermal stability and substrate preference: TrXyn1 has a pI 5.5 and the TrXyn2 has a pI 9, and TrXyn2 is more thermostable and is more active on deacetylated xylan than TrXyn1 [19].

In our earlier work, thermostable xylanases were screened from metagenomic libraries derived from a thermally controlled lignocellulose enriched compost, leading to the discovery of two thermostable xylanases, one of which, Xyl40, was composed of a GH11 catalytic domain (CD) and a carbohydrate-binding domain belonging to family CBM60 [20]. This enzyme was found to be more effective in the hydrolysis of pretreated wheat straw at elevated temperature (55 °C) than TrXyn2 [20]. The aim of the current study was to identify connections between biochemical properties of xylanases and the xylanases resistance towards lignin-caused inhibition by comparing the four structurally similar family GH11 xylanases, TrXyn1, TrXyn2, Xyl40 and the catalytic domain of Xyl40-CD. The effect of lignin and lignin-derived soluble compounds on the three enzymes was followed in hydrolysis and adsorption assays. In addition, thermal stability of the xylanases was defined experimentally, and enzyme surface characteristics were analysed using bioinformatics and modelling.

## Results

### Production and purification of xylanases and lignins

Four xylanases classified to CAZy family GH11 were studied (Table 1). TrXyn1 and TrXyn2 were endogenous proteins of *T. reesei* and were homologously produced. On the contrary, Xyl40, which has been isolated from a metagenomic library and is putatively of prokaryotic origin, was produced heterologously in a prokaryotic host *E. coli* and a eukaryotic host *T. reesei*. The purified *T. reesei* single-domain xylanases TrXyn1 and TrXyn2, and the two-domain Xyl40 produced in *E. coli*, all contained a single major band according to the SDS-PAGE analysis (Additional file 1: Fig. S1). A truncated version Xyl40-CD was produced heterologously in *T. reesei* with the CBM removed. Furthermore, to prevent overglycosylation and consequent misfolding of the putatively prokaryotic protein in the eukaryotic production host, two out of three glycosylation sites were removed from the catalytic domain.

**Table 1:** Characteristics of GH11 xylanases used in the present study

Xylanase	Production host	Domain architecture	Mw (kDa)	pI	pH optimum	Hpatch score			Specific activity (nkat/mg)	Potential salt bridges	Potential disulphide bridges	N-glycosylation
						core	CBM	Total				
TrXyn1	*T. reesei*	GH11	18.4	5.5^a^	4.0–4.5^a^	1.12	–	1.12	5600	3	0	n.d
TrXyn2	*T. reesei*	GH11	18.6	9^a^	5.0–5.5^a^	0.8	–	0.8	23000	5	0	n.d
Xyl40-CD	*T. reesei*	GH11	22.7	8.4^b^	n.d	0.64	–	0.64	13100	5	2	Yes
Xyl40	*E. coli*	GH11-CBM60	35.9	8.6^b^	6.5^c^	0.8	2.24	3.04	3200	5	3	No

*n.d.* not determined in this work

^a^Ref. [19]

^b^Theoretical pI

^c^Ref. [20]

The purification of the single-domain xylanase Xyl40-CD from *T. reesei* culture supernatant resulted in two protein pools. The first pool corresponded to one 23.2 kDa size protein and the second pool corresponded to a mix of a 23.2 kDa and an 18.9 kDa size protein. These accounted for 30% and 70% of the total protein in the second pool, based on the SDS-PAGE band intensities, respectively. Deglycosylation of the second pool of Xyl40-CD with endoH resulted in a disappearance of the upper band, while a single band corresponding to molecular weight of 18.9 kDa remained (Additional file 1: Fig. S2). This indicated that the two protein bands in SDS-PAGE represented glycosylated and non-glycosylated forms of the Xyl40-CD. The glycosylated form of Xyl40-CD in the first pool was used in the following experiments. In addition, the deglycosylated Xyl40-CD was used in studying the role of glycosylation in lignin-derived inhibition.

All the xylanases are classified to family GH11 and had a similar fold in the catalytic domain; however, they differed in several aspects summarised in Table 1. The studied xylanases had a catalytic domain of approximately 20 kDa, but Xyl40, carrying a CBM60 domain, had substantially higher molar mass of 35.9 kDa (Table 1). The CBM60 of bacterium *Cellvibrio japonicus* has been reported to target the binding on xylans, galactans and cellulose through a ligand binding cleft [21]. All xylanases had a low amount of potential salt bridges (3–5). The two forms of Xyl40 had two to three potential disulphide bridges, whereas the TrXyn1 and TrXyn2 did not have any (Table 1). According to Tenkanen et al*.* [19], the *T. reesei* xylanases TrXyn1 and TrXyn2 had only minor amount of structural glycans, < 1% of weight. However, TrXyn1 had no potential N-glycosylation sites, whereas TrXyn2 had three potential glycosylation sites (Fig. 6) and has been reported to be glycosylated [22]. Xyl40 was produced in the prokaryotic host in which glycosylation does not occur. As described above, the production of Xyl40-CD resulted in N-glycosylation of a fraction of the enzyme and the glycosylated fraction was used in the experiments. The deglycosylation of the recombinant Xyl40-CD resulted in a 4.3 kDa reduction in size on the SDS-PAGE. The specific activities on birch wood xylan of the xylanases varied between 3200 and 23,000 nkat/mg protein at pH 5, TrXyn2 having the highest specific activity (Table 1). Previously, TrXyn2 has been observed to have a higher specific activity than TrXyn1 and Xyl40 on birch wood xylan [19, 20]. The genetically engineered Xyl40-CD produced in *T. reesei* had four times higher specific activity on birch wood xylan than the Xyl40 produced in *E. coli*. The presence of glycans can increase or decrease xylanase activity and thermal stability [23, 24]. The higher activity detected with Xyl40-CD may arise not only from glycosylation but also from the different production strains. Proteins may fold incorrectly depending on the production strain, especially correct disulphide bond formation in the periplasm of *E. coli* may not always be successful, resulting in loss of enzyme function [25].

Lignin was isolated from steam pretreated spruce by extensive enzymatic hydrolysis followed by a protease treatment to remove the bound enzymes. The lignin content of the enzymatic hydrolysis residues (EnzHR) was 85.6% with a residual carbohydrate content of 13.9% and nitrogen content of 0.4% after protease treatment (Additional file 1: Table S1). These are within previously reported values of enzymatically isolated lignins [8, 10, 26]. Protease activity was measured from 1% (w/V) EnzHR lignin suspension to confirm that the lignin preparation was not contaminated by proteases used in the isolation procedure. The protease activity of the EnzHR lignin was below the quantitation limit indicating that over 99% of the initially added protease activity had been removed.

### Thermal stability of the GH11 xylanases

**Fig. 1:**
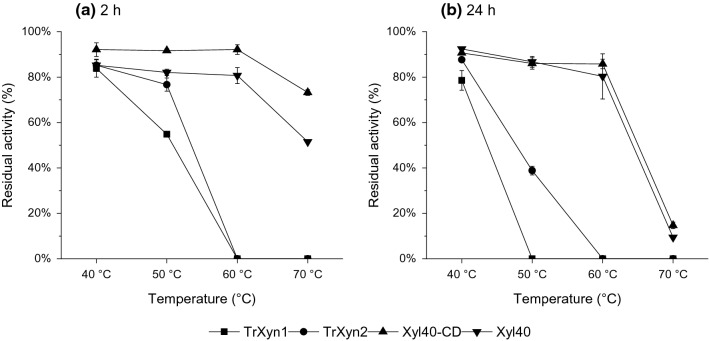
Thermal stability of xylanases. Residual activity of xylanases after **a** 2 h and **b** 24 h incubation at temperatures 40 °C, 50 °C, 60 °C and 70 °C. Activity measurements were performed at 50 °C with the standard xylanase assay before and after incubation

Thermal stability of the enzymes was compared by measuring residual activities of TrXyn1, TrXyn2, Xyl40-CD, and Xyl40 after incubation at 40–70 °C in buffer with 0.1 mg/ml BSA for 2 or 24 h. The metagenomic xylanases Xyl40 and Xyl40-CD had significantly higher thermal stability than the *T. reesei* xylanases (Fig. 1). The metagenomic xylanases retained 80–92% of activity after 24 h of incubation at 40–60 °C and only significantly lost their activity after incubation at 70 °C, having 9–15% residual activity. TrXyn1 and TrXyn2 were both stable at 40 °C, but after 24 h at 50 °C, TrXyn2 retained only 39% of the original activity, whereas the activity of TrXyn1 was totally lost. No residual activity was detected for TrXyn1 or TrXyn2 at incubation for 2 h at 60 °C or above. The measured thermal stabilities of these xylanases were in accordance to previously reported values for TrXyn1, TrXyn2 and Xyl40 [19, 20].

### The effect of lignin on the xylanases

#### Inhibitory effect of lignin on xylan hydrolysis

**Fig. 2:**
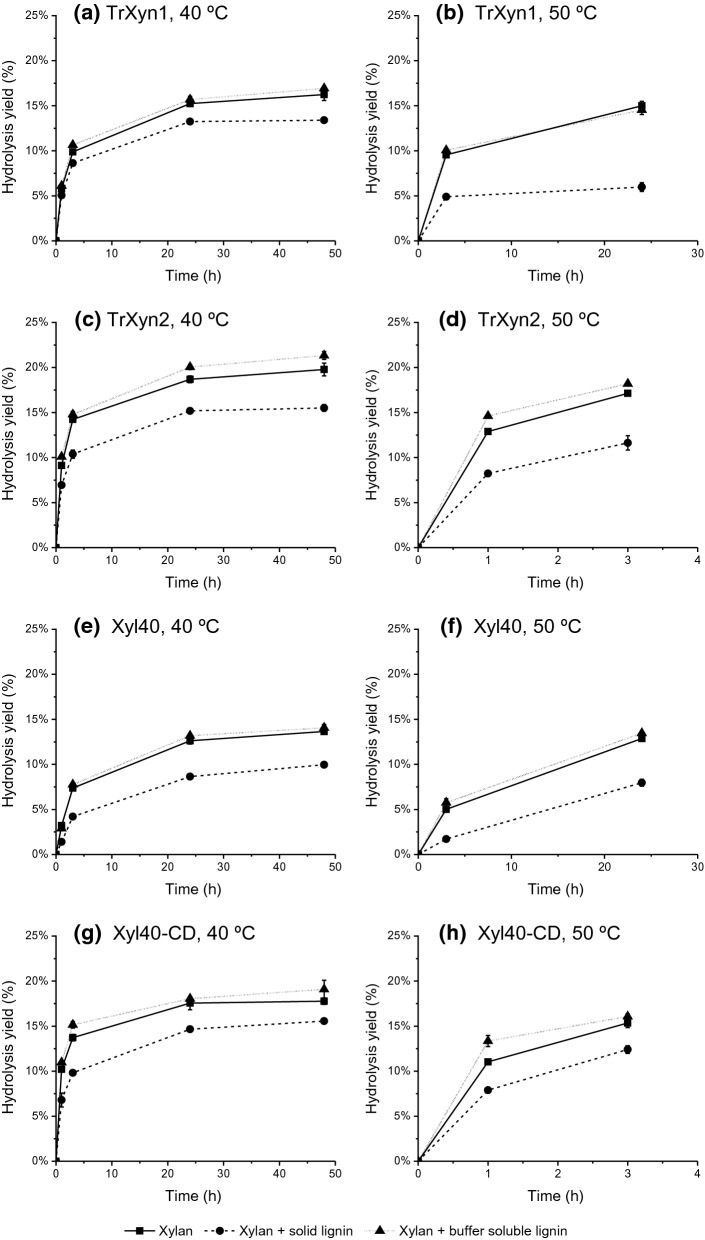
Effect of lignin on xylan hydrolysis at 40 °C (left column) and 50 °C (right column). Hydrolysis of xylan (1% w/V) by xylanases **a**, **b** TrXyn1, **c**, **d** TrXyn2, **e**, **f** Xyl40 and **g**, **h** Xyl40-CD in 50 mM Na-citrate buffer, pH 5. (■) Xylan hydrolysed as such, (●) xylan supplemented with 1% w/V enzymatic hydrolysis residue (EnzHR) lignin from steam pretreated spruce and (▲) xylan supplemented with a buffer soluble fraction of the same lignin. The hydrolysis was followed for 48 h at 40 °C and for 3 h or 24 h at 50 °C

The inhibitory effect of lignin on TrXyn1, TrXyn2, Xyl40-CD and Xyl40 was first compared by carrying out hydrolysis assays with 1% birch wood xylan as substrate in the presence or absence of 1% lignin-rich enzymatic hydrolysis residue (EnzHR) at 40 °C. This temperature was selected based on the measured temperature stabilities of these enzymes (Fig. 1). The enzymes were dosed on protein weight basis (1 µg protein/g dry xylan). TrXyn2 and Xyl40-CD had up to seven times higher specific activity on birch wood xylan than TrXyn1 and Xyl40, which was also seen in the hydrolysis progress curves (Fig. 2, left column). The addition of EnzHR lignin led to a 12–27% decrease in hydrolysis yields compared to the control without lignin for all xylanases (Fig. 2, left column). The most thermostable Xyl40-CD was least affected by lignin.

The effect of temperature on the hydrolysis yields was further studied at 50 °C at selected time points (Fig. 2, right column). At this temperature, the hydrolysis yield of the most thermostable xylanase, Xyl40-CD, was least affected by lignin having only a 19% decrease in yield after 3 h (Fig. 2h). Instead, the hydrolysis yield of the most thermolabile xylanase TrXyn1 was significantly affected by lignin, having a 49% reduction in yield already after 3 h of hydrolysis at 50 °C (Fig. 2b). However, also the yield of the thermostable Xyl40 decreased by 66% after the addition of EnzHR lignin after 3 h (Fig. 2f). TrXyn1 and Xyl40 had a lower hydrolysis rate than TrXyn2 and Xyl40-CD, and therefore, the hydrolysis with these enzymes was carried out to up to 24 h. The hydrolysis yield of TrXyn1 did not increase after 3 h in the presence of EnzHR lignin, confirming the detrimental effect of the lignin on the activity of this enzyme (Fig. 2b). Instead, the hydrolysis reaction with Xyl40 proceeded, and after 24 h the reduction in hydrolysis yield by lignin was only 38% lower compared to the control, indicating that the lignin slowed the xylan hydrolysis but did not completely inhibit the enzyme (Fig. 2f).

Several studies report that hydrolysis residue lignins inhibit enzymes by non-productive binding and inactivating enzymes [6, 7, 10, 11]. Previously, thermostable cellulases have been seen to exhibit lower binding to lignin and increased lignin tolerance in hydrolysis conditions compared to thermolabile cellulases [13]. As such, inhibition of the thermostable xylanase variants Xyl40-CD and Xyl40 even at 40 °C was surprising, suggesting that thermostability is not the only measure for predicting lignin-derived inactivation. Furthermore, the Xyl40 was inhibited by lignin more than Xyl40-CD at the elevated temperature. The major differences between the Xyl40 enzymes were that Xyl40-CD was glycosylated and contained no CBM, whereas Xyl40 was not glycosylated, but had a CBM60 connected to the catalytic domain (Table 1).

#### Effect of glycosylation on xylan hydrolysis

**Fig. 3:**
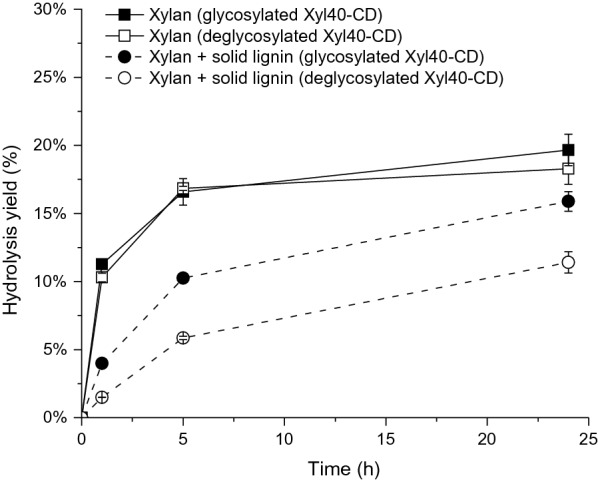
Effect of N-glycosylation of xylanase Xyl40-CD on lignin tolerance. Hydrolysis yield of xylan (1% w/V) (square) and hydrolysis yield of xylan supplemented with 1% EnzHR lignin from steam pretreated spruce (circle). Hydrolysis performed with a glycosylated (filled symbol) and deglycosylated (open symbol) form of Xyl40-CD in 50 mM Na-citrate buffer, pH 5 at 40 °C

The role of N-glycosylation on lignin tolerance was assessed using the enzymatically deglycosylated form of Xyl40-CD. Removal of N-glycans from Xyl40-CD had only a minor effect on the hydrolysis yields on pure xylan but had a remarkable impact on lignin tolerance (Fig. 3). In the presence of EnzHR lignin, the glycosylated form of Xyl40-CD had a 19% lower hydrolysis degree at 24 h when compared to the control without lignin, whereas the non-glycosylated form had a 38% lower hydrolysis degree at 24 h (Fig. 3). This indicates that besides thermal stability, structural glucans improved preservation of the xylanase activity in the presence of lignin.

#### Effect of soluble lignin-derived components on xylan hydrolysis

The role of soluble lignin-derived compounds in the inhibition of xylanases was first studied using a buffer soluble fraction extracted from the EnzHR lignin. The buffer soluble lignin did not inhibit the xylanases confirming that the observed inhibition was due to the solid lignin. Instead, 7–8% increase in the hydrolysis yields was detected for TrXyn2 and Xyl40-CD in the presence of buffer soluble lignin (Fig. 2). These enzymes had higher specific activities than TrXyn1 and Xyl40 (Table 1), both of which were not as clearly affected by the buffer soluble lignin. To further explore the activating effect of the buffer soluble fraction on the xylanases, effects of individual phenolic compounds on the hydrolysis was tested. The compounds were selected based on their presence on lignocellulose pretreatment liquor [27] or in the buffer soluble fraction of isolated lignins [11]. Phenols, protocatechuic acid, syringic acid, ferulic acid, vanillic acid, p-coumaric acid, acetovanillone, vanillin, syringaldehyde and homovanillyl alcohol, were tested at concentrations of 10, 100 and 1000 µg/ml. No activating effects were seen, and only syringaldehyde showed a clear inhibitory effect on the hydrolysis for all the tested xylanases, the effect being most prominent with TrXyn1 with a 50% reduction in the hydrolysis yield (Additional file 1: Table S2). Syringaldehyde was not detected in the buffer soluble fraction of EnzHR lignin isolated from pretreated spruce [11], further confirming that the observed inhibition with EnzHR lignin was due to the solid lignin. Previously, soluble lignin-derived compounds have been seen to be inhibitory to cellulases and xylanases [12, 28]. However, in some cases, low concentrations of monophenols, such as vanillic acid, acetovanillone, protocatechuic acid and ferulic acid, have been reported to increase xylanase activity [16, 29].

#### Xylanase adsorption and inactivation by lignin

**Fig. 4:**
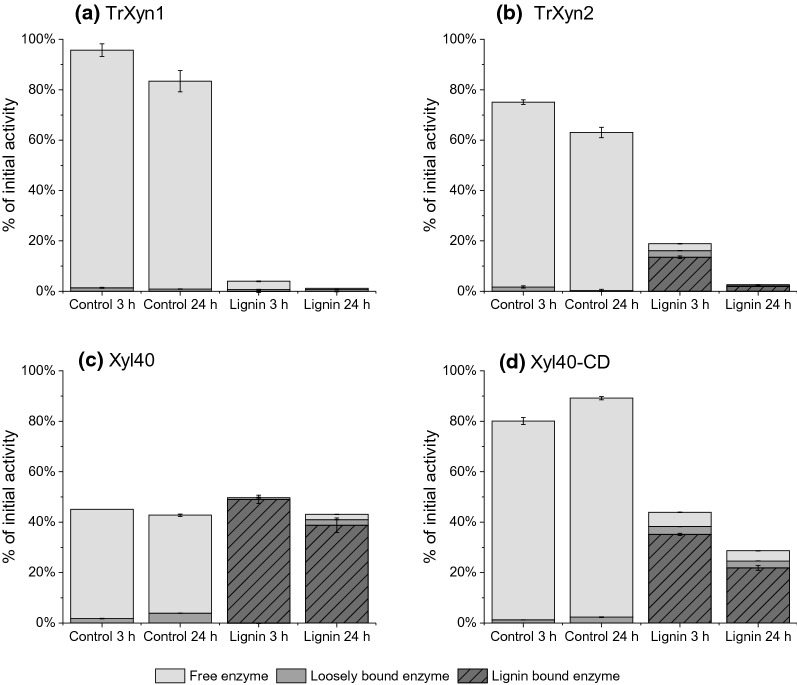
Xylanase activity after incubation with enzymatic hydrolysis residue (EnzHR) lignin. Distribution of xylanase activities for xylanases **a** TrXyn1, **b** TrXyn2,**c** Xyl40 and **d** Xyl40-CD is presented as a percentage of the initially measured activity using the standard assay conditions (50 °C). Xylanase was incubated in 50 mM Na-citrate buffer, pH 5 at 40 °C in the presence of EnzHR lignin isolated from steam pretreated spruce (Lignin) or without lignin (Control). After incubation for 3 or 24 h the solution was centrifuged and the supernatant (Free enzyme) was separated from the solid lignin containing lignin-bound enzymes. The loosely bound enzymes were washed from the lignin with buffer (loosely bound enzyme) and after centrifugation the pellet containing solid lignin was resuspended into buffer for the activity assay (lignin-bound enzyme)

The mechanism of the inhibition by the EnzHR lignin at 40 °C was studied using adsorption experiments. The xylanases were incubated with the EnzHR lignin for up to 24 h. After incubation, the supernatant (free enzyme) and solid fraction (lignin-bound enzyme) were separated by centrifugation. The lignin-bound fraction was washed with buffer containing BSA to remove loosely bound xylanases form the lignin surface (wash buffer). Residual xylanase activity was measured from all fractions and the free and solid fraction were run on SDS-PAGE. Control reactions without lignin were carried out for each of the enzyme to monitor their stability in the test conditions. After incubation with the EnzHR lignin, enzyme activity was lost from the solution indicating that all the xylanases studied were adsorbed to lignin and/or become inactive in the solution (Fig. 4). In case of the thermostable Xyl40-CD and Xyl40, a relatively high proportion of xylanase activity was observed in the solid fraction, comprising 22–49% of the initial xylanase activity added to the reaction, whereas in case of the less thermostable enzymes, TrXyn1 and TrXyn2, all the activity was lost within the 24-h incubation (Fig. 4).

**Fig. 5:**
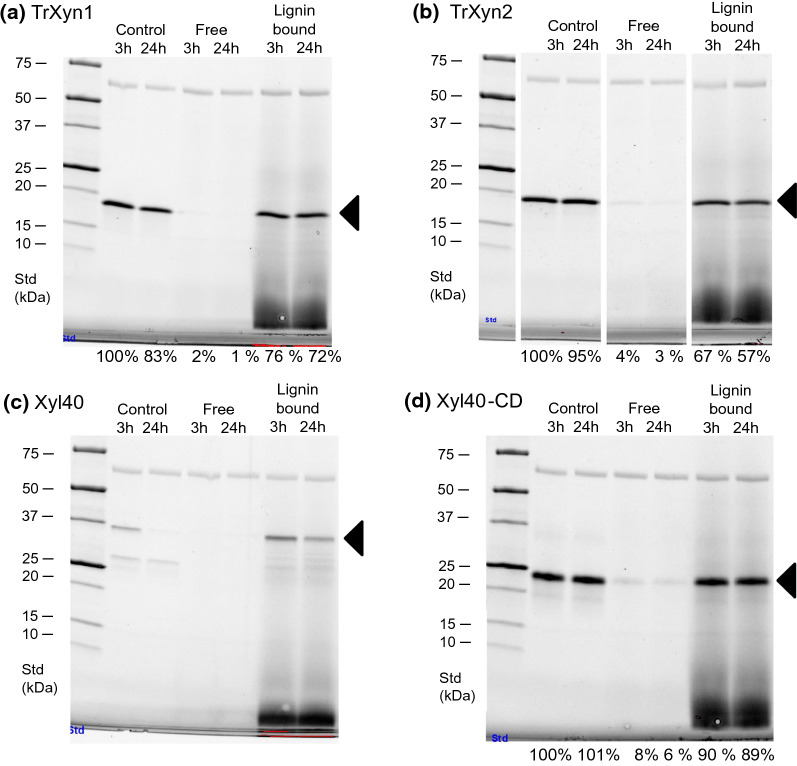
Xylanase distribution after incubation with enzymatic hydrolysis residue (EnzHR) lignin. Distribution of xylanases **a** TrXyn1, **b** TrXyn2, **c** Xyl40 and **d** Xyl40-CD to the supernatant (free enzyme) and solid (lignin-bound enzyme) fractions after incubating 10 µg/ml xylanase with 10 mg/ml EnzHR lignin from isolated from steam pretreated spruce. Enzyme incubated without lignin was used as a control sample. These are same samples as are presented in Fig. 4. Xylanase band is indicated with a triangle and band intensities compared to control as presented as % of control sample at 3 h (% data not shown for Xyl40). A band for BSA, which was added after incubation with lignin, is visible in each sample above the 50 kDa standard. Each xylanase was run on their own gel, but for presentation purposes for **b,** bands were cut to remove unnecessary lanes and indicated as a gap in the image

SDS-PAGE analysis of the free enzyme and lignin-bound enzyme revealed that, indeed, the xylanases TrXyn1, TrXyn2 and Xyl40-CD adsorbed to EnzHR lignin, as the xylanase band was clearly visible in the lignin-bound fraction (57–90% of band intensity) and only a very faint band could be detected in the solution (1–8% band intensity) (Fig. 5). The intensities of the protein bands in the controls without lignin and lignin-bound fractions were very similar for TrXyn1, TrXyn2 and Xyl40-CD, indicating that most of the enzyme could be extracted from lignin using the SDS-PAGE sample buffer. In our earlier work with cellulases, inactivation of the enzymes by lignin adsorption was associated also by impaired release of the enzymes from lignin in the SDS-PAGE analysis, which was interpreted to be due to denaturation of the enzymes on lignin surface [13, 26]. The results suggest that for the xylanases studied here, such irreversible binding was not needed for the enzyme inactivation/inhibition. Unexpectedly, for Xyl40, there was a clear difference in band intensities between 3-h and 24-h samples in the SDS-PAGE (Fig. 5c), while the activity remained constant between 3 and 24 h in the activity assay (Fig. 4c). One explanation for this difference may be that the enzyme produced in *E. coli* was only partially folded properly, *i.e*. all the protein observed on the SDS-PAGE was not composed of active enzyme. Possibly the unfolded protein was sensitive to precipitation in the test conditions and therefore was not detected in the SDS-PAGE at 24 h. However, more research would be needed to confirm these speculations.

In control samples incubated without lignin, TrXyn1 and TrXyn2 retained 80 and 63% of activity, respectively, of the initial xylanase activity for 24 h (Fig. 4a and b). For TrXyn1, the residual activity measured in buffer was in line with the activity measured in buffer with BSA in the thermal stability assay (Fig. 1). Instead, TrXyn2 retained 80% of activity at 40 °C in the thermal stability assay carried out with BSA in the buffer (Fig. 1). There was also a significant difference between Xyl40-CD and Xyl40 in the control reactions: the Xyl40-CD retained most of its activity, 79–87%, over the 24-h incubation at 40 °C, while the activity of Xyl40 declined to 39% within the first three hours. In the thermostability assessment, where the enzymes were incubated at similar conditions, but with 0.1 mg/ml BSA included in the buffer, the Xyl40 was clearly more stable at 40 °C (Fig. 1). The presence of BSA had thus a key role in preservation of the activity of TrXyn2 and Xyl40 in the absence of a substrate. BSA can be added to dilute enzyme mixtures, either to prevent enzyme adsorption onto the tube walls or to stabilise the enzyme. In the adsorption experiment, BSA could not be added to the reaction as BSA is known to bind to lignin reducing the adsorption of other enzymes to lignin surface [30]. In our experiments low protein binding tubes were used instead.

### Comparison of structural features of the GH11 xylanases

**Fig. 6:**
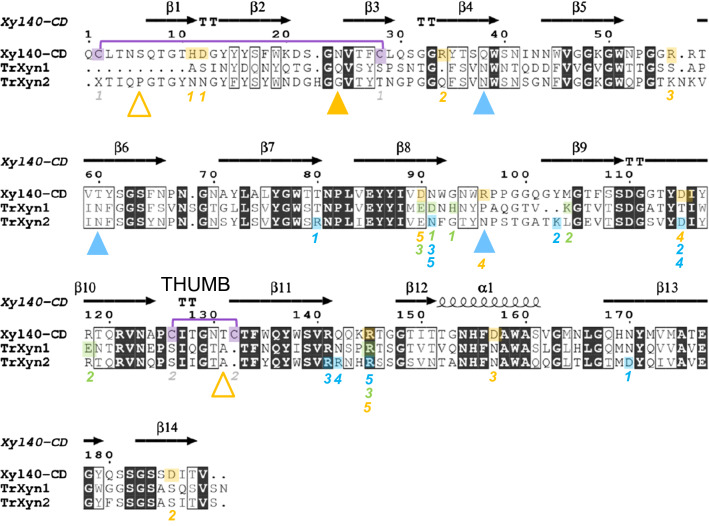
Structure-based sequence alignment of Xyl40-CD, TrXyn1 and TrXyn2 performed with the ESPrit web server. Secondary structural elements of the Xyl40-CD are indicated above the alignment (β-strand arrows and α-helices). Strictly identical amino acid residues are marked in white letters on a black background. Regions of conserved, highly similar residues are framed in thin-lined boxes with bold letters. Possible cysteine bridges are highlighted in purple. Possible salt bridges are highlighted in orange for Xyl40-CD, green for TrXyn1 and blue for TrXyn2; predicted N-glycosylation sites are marked as triangles in orange for Xyl40-CD (empty triangles correspond to removed glycosylation sites of Xyl40-CD) and blue for TrXyn2

The two GH11 xylanases from *T. reesei* and the two variants of the metagenomic xylanase Xyl40 were computationally characterised focusing on structural features possibly affecting thermal stability, adsorption to lignin and activity in the presence of lignin. The analysis was done on levels of amino acid sequence and tertiary structures. The metagenomic xylanase Xyl40-CD shared 43% sequence identity with TrXyn1 and 53% with TrXyn2. GH11 xylanases have a highly conserved structure comprising two β-sheets packed against each other forming a shape described as a “right hand” [31]. Structure-based sequence alignment of the catalytic domains of TrXyn1, TrXyn2 and Xyl40-CD showed that the conserved areas in the protein sequences (Fig. 6) were located in the β-strands in the palm region, at the catalytic site as well as the α-helix on the outer surface and C-terminal end in the finger region.

#### Disulphide bridges and salt bridges

**Fig. 7.**
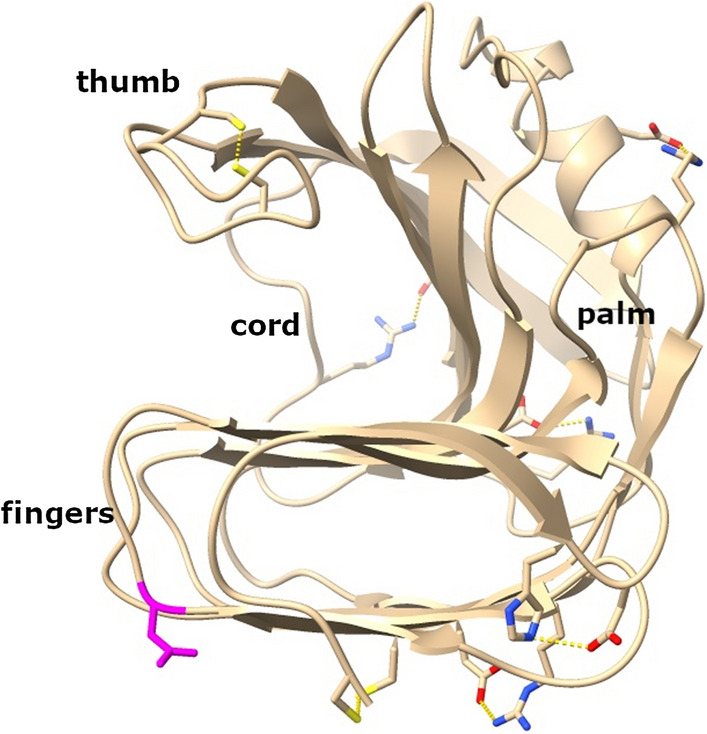
3D structural model of Xyl40-CD. Predicted N-glycosylation site Asn24 in magenta, two potential disulphide bond sites (Cys2-Cys28 and Cys126-Cys132) in stick form in yellow, five potential salt bridges in stick form with positive sites in blue and negative in red. Visualised with ChimeraX

There was a clear difference detected between the mesophilic *T. reesei* xylanases and the thermophilic Xyl40-CD in the amount of cysteine (Cys) residues. The catalytic domain of Xyl40-CD contains two pairs of Cys residues and the CBM60 of Xyl40 a third pair, all pairs within 2.03 Å distance and as such, likely sites for disulphide bridges. On the contrary, TrXyn1 and TrXyn2 sequences did not contain any Cys residues. The Cys-pairs in the 3D model of Xyl40-CD were located in the N-terminal region and thumb region (Fig. 7). A similar disulphide bridge in the N-terminal region has been found in the catalytic domain of family GH11 xylanase *Ev*Xyn11. The Xyl40 xylanase originates from an uncultured bacterium and is identical to the PDB template sequence of *En*Xyn11A used for modelling Xyl40-CD [32]. The 15 min half-life of *Ev*Xyn11 has been reported to be improved from 64 °C to 89 °C after seven mutations in the N-terminal region [32], thus highlighting the importance of the N-terminal region for improved thermal stability of GH11 xylanases.

The presence of salt bridges between negatively or positively charged amino acids in the xylanase structures was assessed using an ESBRI web server tool. The predicted number of salt bridges in xylanases TrXyn1, TrXyn2 and catalytic domain of the Xyl40 varied between 3 and 5 (Table 1). Interestingly, the location of the predicted salt bridges was very different in the three xylanases, except for the conserved salt bridge [33] buried in the enzyme core (Fig. 6). In Xyl40-CD, there were two distinct salt bridges, one between the α-helix and palm region and the other close to the C-terminal in the finger region (Fig. 7). The α-helix and N-terminal regions have been suggested to be the initiation sites for thermal unfolding for GH11 xylanases based on molecular dynamic and mutagenesis studies [34]. Stabilisation of these regions with salt bridges can potentially increase the thermal stability of the enzyme. Although the stabilising effect of salt bridges has not been as clearly demonstrated as in the case of disulphide bridges, a higher number of salt bridges are often found in more thermostable enzymes than in their mesophilic counterparts [33].

#### Surface hydrophobicity and charge

The surface hydrophobicity of the xylanases was analysed with Rosetta protein modelling software. Here, each enzyme is given a hydrophobic patch (Hpatch) score, which is an indicator of larger uniform hydrophobic patches on the protein surface. The Hpatch scores for the xylanases varied between 0.64 and 3.04, with the highest individual score of 2.24 going to the CBM60 of Xyl40 (Table 1). For comparison, Hpatch scores for carbohydrate active enzymes have been previously reported to vary between 0.8 and 2.8 for xylanases, 7.0 and 16.0 for endo- and exocellulases, and 17.9 and 45.9 for β-glucosidases [9, 11]. The analysed xylanases had low surface hydrophobicity compared to cellulases and β-glucosidases. Previously, Sammond et al. [9] have observed a correlation between high Hpatch score and protein binding to lignin [9].

**Fig. 8:**
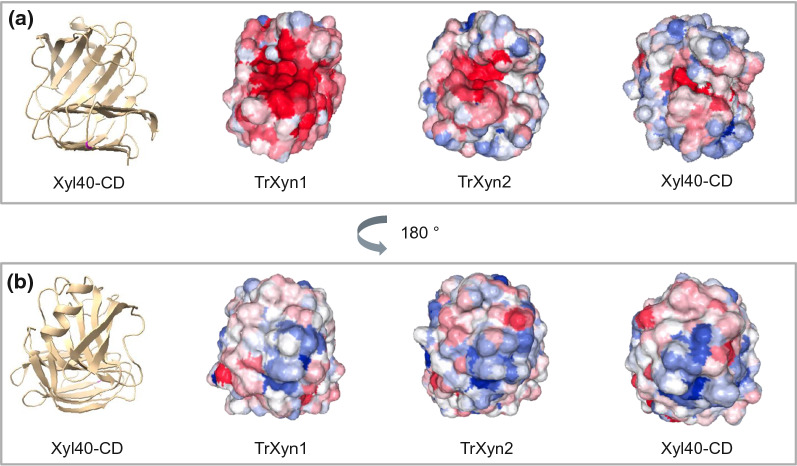
Surface charge distribution on xylanases TrXyn1, TrXyn2 and Xyl40-CD calculated with Protein-Sol web tool. Charge at pH 6.3 **a** from the front of the active site palm region and **b** turned 180° from the back of the palm region. Cartoon image on Xyl40-CD to represent protein orientation. Negatively charged areas are shown in red and positively charged in blue

All the xylanases had a pI of over 5, indicating that they carried a net positive charge under the hydrolysis pH 5 (Table 1). The xylanases Xyl40, Xyl40-CD and TrXyn1, with pIs ranging from 8.4 to 9, carried a higher positive charge than TrXyn1, which had a pI of 5.5. The pI is an indication of the net surface charge, but the local charge distribution on the enzyme surface may vary more significantly. The local surface charges of enzymes was modelled using the Protein-Sol web tool [35]. The Protein-sol patches algorithm was developed to predict protein solubility in buffer but has been expanded to calculate hydrophobic patches and electric charge distribution on the protein surface at pH 6.3. Since the catalytic domains of Xyl40 and Xyl40-CD were nearly identical in terms of amino acid sequences, only Xyl40-CD was used in the analysis and compared to TrXyn1 and TrXyn2. As expected, the enzymes TrXyn2 and Xyl40 had more positively charged areas on the protein surface compared to TrXyn1. The active sites of all the three xylanases were negatively charged, but TrXyn1 had a significantly larger negatively charged area surrounding the active site cleft (Fig. 8a). Interestingly, all xylanases had a positively charged area in the back of the palm region, on the opposing side of the catalytic cleft (Fig. 8b).

#### Amino acid composition and N-glycosylation sites

Comparison of the amino acid composition (Table 2) revealed that the Xyl40-CD had features similar to other known thermostable GH11 xylanases [33]. The catalytic domain of Xyl40-CD had higher threonine (Thr) to serine (Ser) ratio (1.31) than TrXyn1 (0.78) or TrXyn2 (0.73). High Thr/Ser ration is proposed to improve β-sheet formation leading to more rigid fold [33]. The Xyl40-CD also had relatively high arginine and tryptophan content and low valine content, all of which have been found to be characteristic for thermostable xylanases [33]. The catalytic domain of Xyl40 had three predicted N-glycosylation sites of which two were removed from the engineered Xyl40-CD produced in *T. reesei*. The remaining N-glycosylation site Asn24 was located in the finger region of Xyl40-CD (Figs. 6 and 7). Xyl40 was produced in *E. coli* and as such was not glycosylated. TrXyn1 had no predicted N-glycosylation sites and TrXyn2 had three potential N-glycosylation sites (Fig. 6).

**Table 2:** Amino acid composition and Thr/Ser ratio of xylanases TrXyn1, TrXyn2 and Xyl40-CD

	TrXyn1	TrXyn2	Xyl40-CD
Percentage of amino acids (%)			
Ala	5.1	3.7	3.2
Arg	1.7	3.2	4.2
Asn	10.1	10.5	9.0
Asp	2.8	2.1	3.7
Cys	0.0	0.0	2.1
Gln	6.2	5.3	5.8
Glu	2.8	2.1	1.1
Gly	12.4	14.2	14.8
His	1.7	1.6	1.6
Ile	3.4	4.7	3.2
Leu	3.4	2.6	3.2
Lys	0.6	2.1	1.6
Met	1.1	0.5	2.1
Phe	3.4	4.2	3.2
Pro	3.4	3.7	3.2
Ser	12.9	11.6	8.5
Thr	10.1	8.4	11.1
Trp	3.4	3.2	5.3
Tyr	5.6	8.9	7.9
Val	10.1	7.4	5.3
Thr/Ser ratio	0.78	0.73	1.31

## Discussion

### Structural features of xylanases affecting enzyme binding and inactivation on lignin

Lignin had an inhibitory effect on all the GH11 xylanases studied in this work; furthermore, inhibition was associated to binding of the enzymes on solid lignin surface and partial or total inactivation of the enzyme. At 40 °C, TrXyn1 was not significantly affected by lignin in the hydrolysis experiment with only a 17% reduction in hydrolysis yield but was the first enzyme to lose activity on the lignin surface in the binding studies. The substrate had thus a protecting effect on the TrXyn1 activity in the presence of lignin. This was possibly due to preferred adsorption on xylan over lignin. The presence of substrate has also been seen to increase the thermal stability of a GH10 xylanase [23].

At higher temperature, 50 °C, the effect of lignin in hydrolysis assays was most detrimental on the least thermostable enzyme TrXyn1, and the most thermostable enzyme Xyl40-CD was least affected. High thermal stability has been connected to higher lignin tolerance with cellobiohydrolases [13]. Adsorption of a thermolabile TrCel7A to lignin has been shown to result in a rapid inactivation of the enzyme [26]. On the contrary, a more thermostable *Talaromyces emersonii* cellobiohydrolase, having almost a 10 °C higher melting temperature, T_m_, than TrCel7A, has been shown to be essentially unaffected by lignin at 45 °C [13]. Non-productive adsorption of cellulases on lignin is especially detrimental to the enzyme as cellulases act on the solid cellulose substrate and need to be able to move to the substrate. The Xyl40-CD studied here was stable at 60 °C for 24 h, but its hydrolytic activity was clearly impaired by lignin already at 40 °C. The Xyl40-CD and Xyl40, however, partially retained their activity when bound on lignin, which can be due to the more rigid structure and consequent resistance towards unfolding on the lignin surface. In case of a soluble substrate, such as xylan used in this study, binding of enzyme on lignin may not prevent the catalysis, if the active site remains accessible. Previously, β-glucosidases and xylanases from a commercial cellulase cocktail Cellic CTec2, both of which act on a soluble substrates, have been reported to bind to lignin, but remain active on the lignin surface [15]. Enzyme immobilisation on a carrier can potentially increase enzyme stability and catalytic activity and enable re-use of the enzyme. The use of lignin nanoparticles has even been studied as a low-cost biodegradable alternative for enzyme immobilisation [36].

In the current study, the glycosylated form of the thermally stable Xyl40-CD was clearly more lignin tolerant than the deglycosylated form, indicating that the glycan(s) had a protective effect towards lignin inhibition. The potential glycosylation site in Xyl40-CD was located in the finger region and did not interfere with the active site. Glycosylation has been reported to either enhance or decrease the thermal stability of GH10 or GH11 xylanases [23, 24]. The role of enzyme glycosylation on lignin tolerance of enzymes has been studied less. Recently, it has been reported that deglycosylation of *T. reesei* Cel7A slightly increases the non-productive adsorption of this cellobiohydrolase to lignin [37]. Furthermore, by changing the glycosylation pattern of the CBM1 of TrCel7A, preferential adsorption to cellulose over lignin could be increased [38]. The hydrolytic performance of GH11 xylanases in the presence of lignin could thus potentially be improved by introducing N-glycans to the catalytic domain, as long as the glucans do not interfere with the catalytic site.

In the adsorption assay, all the xylanases adsorbed to lignin in the experimental conditions used. The adsorption of cellulases on lignin surfaces has been postulated to be driven both by electrostatic and hydrophobic interactions [9, 10, 39]. The hydrophobic patch scores of the studied xylanase were low compared to cellulases and β-glucosidases [9], suggesting that the hydrophobic interactions were not driving the adsorption of these xylanases. Xylanases in the commercial enzyme cocktail Cellic CTec2 have been reported to adsorb onto milled wood lignin from pretreated wheat straw by electrostatic interactions [15]. EnzHR lignin from spruce has a negative charge at pH 5 [40], which promotes the binding of positively charged enzymes [10, 11, 40]. The TrXyn1 had a low pI 5.5, whereas xylanases TrXyn2, Xyl40 and Xyl40-CD had a much higher pI 8–9. Consequently, under the hydrolysis conditions at pH 5 the xylanases TrXyn2, Xyl40 and Xyl40-CD were positively charged, while the overall charge of TrXyn1 was expected to be closer to neutral. Therefore, the overall charge of the xylanases could not explain the similar binding to lignin. The distribution of charged residues on the surfaces of the TrXyn1, TrXyn2 and Xyl40 catalytic domains was studied using the crystals structures and homology models. Common for all the xylanases studied here was a region rich in positively charged residues located at a loop close to the α-helix on the opposite side of the active site, while the active site was negatively charged. The positively charged areas on the enzyme surface may be potential binding sites to the negatively charged lignin. On the other hand, we have previously observed that a *T. reesei* endoglucanase TrCel7B, which has a pI 4.5–4.9 and does not contain large hydrophobic patches, exhibits high affinity towards lignin at pH 5 [11] and forms aggregates on the lignin surface [41]. Proteins with a neutral net charge lack repulsive forces that are present between similarly charged enzymes and can be more prone to aggregate on hydrophobic surfaces [42]. The close to neutral net charge of TrXyn1 could also increase binding to the partially hydrophobic lignin.

The CBM1 of *T. reesei* cellulases increases cellulase adsorption to lignin [7, 39]. The adsorption occurs at least partially via the planar cellulose binding surface of the CBM1 and binding is thought to be mediated by π–π stacking of the aromatic amino acids on the planar surface of the CBM1 and aromatic moieties on the lignin surface [40]. Instead, the CBM60 in Xyl40 did not have clear effect on the binding to the EnzHR lignin. The CBM60 has a binding cleft as opposed to a flat binding surface [21], which may affect the CBM60’s affinity to lignin. This notion is supported in a study by Zhang et al*.* [43], who observed that a CBM from family 2b with a non-planar binding surface did not increase xylanase binding to lignin [43].

Protein binding to cellulose requires a specific cellulose binding surface, whereas binding to lignin has been proposed to be less specific [44, 45]. Partial inactivation of xylanases may have been due to non-specific binding via different sites on the enzyme surface, leading to inactivation of a fraction of the enzyme. Alternatively, binding via one binding site could result in lignin restricting the movement of the enzymes and consequently a lower enzyme activity, however, retaining the enzyme structure.

### Structural features affecting thermal stability

The metagenomic Xyl40 xylanases, with and without a CBM, were more thermostable than the xylanases TrXyn1 and TrXyn2 originating from *T. reesei*. The structural features affecting the thermal stability of GH11 xylanases have been studied earlier in molecular dynamic (MD) studies, comparing 3D structures of thermostable and mesophilic xylanases and by engineering the thermal stability of xylanases [33, 34, 46]. MD studies on seven mesophilic and five thermophilic family GH11 xylanases suggest that the thermal unfolding pathways are similar for the studied xylanases, independent of the enzyme thermostability, and that the enzyme unfolding initiates either at the N-terminal region or at the α-helix region and then continues to the finger region [34]. Supporting the MD simulation studies, the introduction of disulphide bridges especially to the N-terminal and alpha-helix regions has been shown to increase the thermal stability of TrXyn2 by over 20 °C [46]. The potential N-terminal Cys2-Cys28 disulphide bridge in the catalytic domain of Xyl40 was thus likely to contribute to the increased thermal stability of this xylanase. In addition, the salt bridge in the α-helix region could stabilise the enzyme. By contrast, the thumb region has a lot of movement in molecular simulations, but it is not a location of protein unfolding [34]. Mobility of the thumb region is thought to be essential for enzyme function [47]. Possibly stabilising the labile thumb region with a Cys126-Cys132 disulphide bond in Xyl40 could increase the optimal temperature of the enzyme by reducing excess movement of the thumb region in high temperatures. The predicted roles of the potential disulphide and salt bridges should be confirmed with mutation studies for conclusive results.

## Conclusions

All the studied GH11 xylanases, TrXyn1, TrXyn2, Xyl40 and Xyl40-CD, were adsorbed and inhibited by enzymatically isolated lignin from steam pretreated spruce. However, the two forms of the thermostable Xyl40 enzyme could partially retain their activity when adsorbed to the lignin surface. N-glycosylation of Xyl40-CD increased the lignin tolerance of the xylanase. Thermostability of Xyl40 was most likely achieved by stabilising the protein areas that are most prone for unfolding. The higher stability may have been achieved by a putative disulphide bond in the N-terminal region and salt bridge in the α-helix region. The binding of TrXyn2 and the two Xyl40 enzymes on lignin was most likely driven by electrostatic interactions, while for TrXyn1 other mechanism may be involved. Understanding the mechanism behind enzyme adsorption and inactivation by lignin can aid in developing more efficient enzymes for biomass deconstruction.

## Methods

### Lignin isolation and characterisation

Steam pretreated spruce (5 min at 215 °C with 3 wt-% SO_2_) was kindly provided by Lund University, Sweden [48] and enzymatic hydrolysis residue (EnzHR lignin) was isolated from the biomass by extensive enzymatic hydrolysis using 20 FPU/g dry matter (DM) Celluclast 1.5 L supplemented with 500 nkat/g DM Novozym 188 (Novozymes, Bagsværd, Denmark) followed by a protease treatment [26]. Briefly, hydrolysis was conducted at 6% (w/v) DM content, 45 °C, 50 mM Na-acetate, pH 5, with 0.02% of NaN_3_ and fresh enzyme and buffer was replaced every 24 h. After 72 h of hydrolysis the residual solids were washed three times with pH-adjusted mQ water (pH 2.5). To remove potential enzymes attached to the residual solid, a bacterial type (XXIV) protease (Sigma, Japan) was added at 1 mg protease/50 mg lignin and incubated with mixing for 24 h at 37 °C in 0.5 M NaHCO_3_, pH 9.6, 5% w/v DM. The solid residue was again washed with pH 2.5 water and freeze dried. A buffer soluble fraction of the EnzHR lignin was prepared by incubating 2% (w/V) EnzHR lignin in 50 mM Na-citrate buffer pH 5 mixing overnight and separated from the solid fraction by centrifugation. The carbohydrate, lignin and nitrogen content of the EnzHR lignin were analysed as described in Ref. [8]. The residual protease activity of the isolated EnzHR lignin was measured with the commercial Azocasein (Megazyme, Ireland) assay according to the manufacturer’s instructions in ½ scale. For the assay, 1% (w/V) EnzHR lignin was suspended in 50 mM Na-citrate buffer pH 5.

### Enzyme production and purification

The *Trichoderma reesei* xylanases TrXyn1 (pI 5.5, also named TrXyn11B) and TrXyn2 (pI 9, also named TrXyn11A) were homologously produced and purified from a *T. reesei* culture supernatant according to Ref. [19]. A metagenomic xylanase Xyl40, with most likely prokaryotic origin, was produced in XL-1 Blue *E. coli* (Stratagene) and purified from the periplasm according to [20] with slight modifications, *i.e.* the production was performed at 37 °C and protein production induced with tetracycline. The xylanase produced in *E. coli* has a carbohydrate-binding module (CBM60) and in this article the enzyme is referred to as Xyl40. A truncated version of Xyl40, lacking the CBM60, was kindly produced in an engineered *T. reesei* strain by ROAL Ltd (Rajamäki, Finland) and is referred to as the catalytic domain of Xyl40 (Xyl40-CD). To prevent overglycosylation and misfolding of the presumably prokaryotic protein in the eukaryotic production host *T. reesei*, the CBM60 was removed and two potential N-glycosylation sites were removed from the catalytic domain by making the following amino acid mutations in the GeneBank AWD75450 sequence: Asn6Ser and Asn131Thr. The culture supernatant for Xyl40-CD was heat treated for 2 h at 60 °C, in 100 mM Na-citrate buffer pH 5. To remove impurities anion exchange chromatography (DEAE Sepharose FF, GE Healthcare) in 10 mM phosphate buffer pH 7.2 was used and the xylanase was recovered from the flow-through. Further purification was performed with cation exchange chromatography (5 ml HiTrap SP, GE Healthcare) in 50 mM Na-acetate buffer pH 5 and the xylanase was eluted with 0 to 1 M NaCl gradient producing two pools of enzymes. Pool 1 contained a 23.2 kDa molecular weight protein and pool 2 contained a mixture of a 23.2 kDa and an 18.9 kDa sized proteins.

### Xylanase characterisation

The molecular weight was estimated using precast sodium dodecyl sulphate–polyacrylamide gel electrophoresis (SDS-PAGE) gels (Bio-Rad, USA) and visualised using the Molecular Imager, Gel Doc XR (Bio-Rad, USA). The standard xylanase activity assay used in this work was performed with 1% birch wood xylan (Carl Roth GmbH, Karlsruhe, Germany) in 50 mM Na-citrate buffer, pH 5 for 5 min at 50 °C [49]. Thermal stability was measured by incubating 10 µg/ml of enzyme at temperatures 40, 50, 60 and 70 °C for 2 h or 24 h in 50 mM Na-citrate buffer, pH 5 with 0.1 mg/ml bovine serum albumin (BSA). The residual activity was measured using the standard activity assay with dilutions made into buffer containing 0.1 mg/ml BSA. The N-glycans in Xyl40-CD were removed by endoH treatment (New England Biolabs) in non-denaturing conditions: 20 µg of protein with 1 µl of endoH was incubated in 50 mM Na-citrate buffer, pH 5 at 37 °C for 30 min, and run on SDS-PAGE to estimate the size of proteins. All enzyme characterisation measurements were performed in protein low binding tubes (Protein LoBind, Eppendorf) to prevent adsorption of protein to the plastic surface and loss of activity due to the low protein concentration used in many experiments.

### Computational analysis of xylanase sequences and structures

The theoretical isoelectric point and amino acid composition of the xylanases were calculated based on the protein sequences (without predicted signal sequences) using the Expasy ProtParam tool (https://web.expasy.org/protparam/) [50]. Structural comparison was carried out using the crystal structures 1XYN and 1ENX [31] for TrXyn1 and TrXyn2, respectively, downloaded from the Protein Data Bank (https://www.rcsb.org/)www.rcsb.org [51]. For the catalytic domains of Xyl40 and Xyl40-CD, 3D models were built using Swiss-model homology modelling tool (https://swissmodel.expasy.org/) [52] using PDB structure 2VGD, derived from an environmental metagenomic library GH11 xylanase *En*Xyn11A, as template [53]. *En*Xyn11A had 80% sequence identity to Xyl40-CD. The CBM of Xyl40 was modelled on PDB structure 2XHH, a CBM60 of *Cellvibrio japonicus* GH11 xylanase [21]. These two CBMs shared 61% sequence identity. The hydrophobic patch (Hpatch) score and potential disulphide bonds were calculated using the enzyme design software Rosetta (https://www.rosettacommons.org) [54]. N-glycosylation sites were predicted using NetNGlyc server [55]. Structure-based sequence alignment was performed using ESPrit web server with default parameters (http://espript.ibcp.fr) [56]. Potential salt bridges were calculated using ESBRI web server (http://bioinformatica.isa.cnr.it/ESBRI/) [57]. The local surface charges of the xylanases were calculated using Protein-sol patches algorithm (manchester.ac.uk) [35]. Visualisation of protein structures was performed with UCSF ChimeraX software developed by the Resource for Biocomputing, Visualization, and Informatics at the University of California, San Francisco [58].

### Assays for xylan hydrolysis and inhibitory effect of lignin

Xylan (1% w/V) was hydrolysed with 1 µg/ml of xylanase in the presence or absence of (i) EnzHR lignin from steam pretreated spruce (1% w/V) or (ii) a buffer soluble fraction of EnzHR lignin in a concentration corresponding to the concentration to buffer soluble lignin in experiment (i). Background controls used were xylan, lignin and enzyme with lignin. Hydrolysis was performed in triplicate in 50 mM Na-citrate buffer, pH 5 in 40 or 50 °C for up to 48 h with mixing in 200 µl volume in protein low binding tubes (Protein LoBind, Eppendorf). Reducing sugars were analysed using the PAHBAH assay [59] after terminating the reaction by incubation at 98 °C for 10 min. The effect of monomeric phenolic compounds on the hydrolysis of xylan was analysed in similar conditions as described above, but instead of lignin, a phenolic compound, protocatechuic acid, syringic acid, ferulic acid, vanillic acid, *p*-coumaric acid, acetovanillone, vanillin, syringaldehyde or homovanillyl alcohol, was added to the reaction in 10, 100 or 1000 µg/ml concentration and a protein low binding 96-well plate (Protein LoBind, Eppendorf) was used.

### Adsorption experiments

Adsorption of the xylanases on lignin was studied by incubating 50 µg/ml of xylanase with 1% (w/V) EnzHR lignin in 50 mM Na-citrate buffer, pH 5, at 40 °C in 400 µl volume with mixing 1400 rpm. Each reaction was carried out in triplicates. Aliquots of 150 µl were taken after 3 and 24 h and centrifuged 12,000 *g* 5 min to separate the solid and liquid fractions. The supernatant (free enzyme) was immediately supplemented with BSA in a concentration of 0.1 mg/ml to improve stability of the xylanases. Buffer containing 0.1 mg/ml BSA (150 µl) was added to the solid fraction to release loosely bound enzymes from the lignin surface. The sample was centrifuged and supernatant separated (loosely bound enzyme). Prior to analysis, 150 µl of buffer with BSA was added to the solid fraction (lignin-bound enzyme). Residual enzyme activity was analysed from all three fractions with the standard xylanase assay at 50 °C. The supernatant and solid fraction were analysed with SDS-PAGE using a method modified from [26]. The two fractions were run on 4–20% Tris–HCl 1.0 mm Criterion precast SDS-PAGE gels (BioRad) by mixing the sample with ¼ running buffer and loading 25 µl of undiluted sample to the gel.

## Supplementary Information

**Additional file 1:** **Figure S1.** SDS-PAGE image of purified proteins. **Figure S2.**SDS-PAGE image of endoH treatment to Xyl40-CD. **Table S1.** The carbohydrate, lignin and nitrogen composition of enzymatic hydrolysis residue lignin. **Table S2.** Effect of soluble phenolic compounds on xylan hydrolysis by xylanases TrXyn1, TrXyn2, Xyl40 and Xyl40-CD.

## Data Availability

All data generated during this study are included in the article and additional files. Raw data used and/or analysed during the current study are available from the corresponding author on reasonable request.

## Abbreviations

CBM: Carbohydrate-binding module
CD: Catalytic domain
EnzHR: Enzymatic hydrolysis residue
GH: Glycoside hydrolase
PAHBAH: 4-Hydroxybenzoic acid hydrazide
SDS-PAGE: Sodium dodecyl sulphate–polyacrylamide gel electrophoresis
