# Supplementary material for: Inhibitory effect of lignin on the hydrolysis of xylan by thermophilic and thermolabile GH11 xylanases
Source: Biotechnol Biofuels Bioprod. 2022 May 14;15:49. doi: 10.1186/s13068-022-02148-4 (PMC9107766; doi:10.1186/s13068-022-02148-4)
Supplement: Supplementary file 1 — Additional file 1: Figure S1. SDS-PAGE image of purified proteins. Figure S2.SDS-PAGE image of endoH treatment to Xyl40-CD. Table S1. The carbohydrate, lignin and nitrogen composition of enzymatic hydrolysis residue lignin. Table S2. Effect of soluble phenolic compounds on xylan hydrolysis by xylanases TrXyn1, TrXyn2, Xyl40 and Xyl40-CD. [file 13068_2022_2148_MOESM1_ESM.pdf]

Supplementary Material for:

Inhibitory effect of lignin on the hydrolysis of xylan by thermophilic and thermolabile GH11 xylanases

Miriam Kellock[a], Jenni Rahikainen[a], Anna S. Borisova[a], Sanni Voutilainen[a], Anu Koivula [a], Kristiina Kruus[b] and Kaisa Marjamaa[a]

[a]VTT Technical Research Centre of Finland Ltd, P.O Box 1000, 02044 VTT, Finland
[b]Aalto University, P.O. Box 16100, 00076 Aalto, Finland

Table of Contents

Fig. S1. SDS-PAGE of purified xylanases. 2

Fig S2. SDS-PAGE analysis of deglycosylation of Xyl40-CD. 3

Table S1. Composition of the enzymatic hydrolysis residue (EnzHR) lignin isolated from steam pretreated spruce. 4

Table S2. Effect of soluble phenolic compounds on the hydrolysis yields of xylan by xylanases TrXyn1, TrXyn2, Xyl40-CD and Xyl40. 5

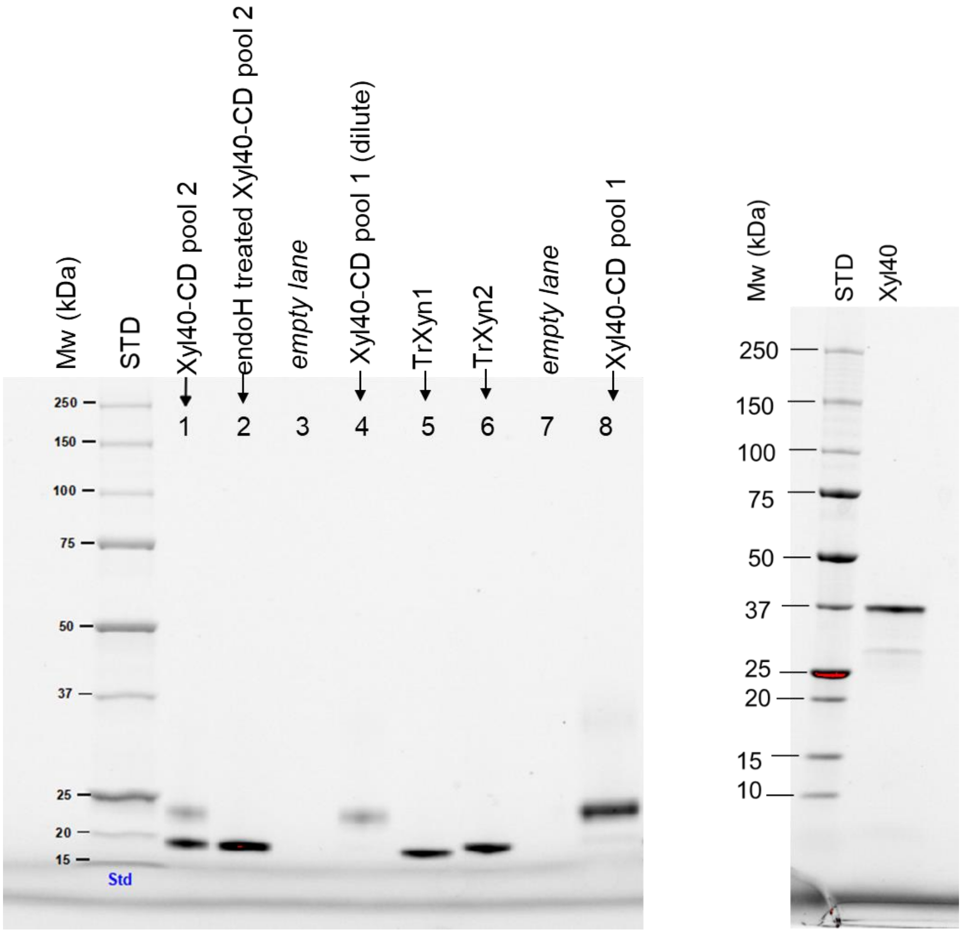

Fig. S1. SDS-PAGE of purified xylanases: TrXyn1 (left gel, lane 5), TrXyn2 (left gel, lane 6), Xyl40 (right gel), Xyl40-CD pool 1(left gel, lane 8) and Xyl40-CD pool 2 (left gel, lane 1). TrXyn1, TrXyn2 and the two pools of Xyl40-CD were run on one gel and Xyl40 on a separate gel. For comparison, the same molecular weight standard (STD) was run on both gels.

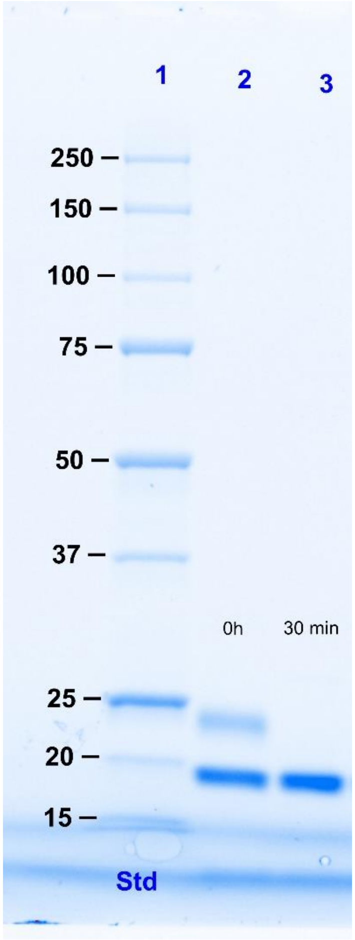

Fig S2. SDS-PAGE analysis of deglycosylation of Xyl40-CD.
Lane 1, molecular weight standard. Lane 2, Pool 2 from Xyl40-CD purification. Lane 3, Pool 2 from Xyl40-CD purification after incubation with endoH for 30 min.

Table S1. Composition of the enzymatic hydrolysis residue (EnzHR) lignin isolated from steam pretreated spruce.

	Glucose	Xylose	Mannose	Total polysaccharides	Acid soluble lignin	Acid insoluble lignin	Nitrogen
EnzHR	13.2	0.3	0.4	12.8	0.6	85.0	0.4

Presented as % (w/w) of dry matter.

Table S2. Effect of soluble phenolic compounds on the hydrolysis yields of xylan by xylanases TrXyn1, TrXyn2, Xyl40-CD and Xyl40. Yield are presented as % out of the control sample without a phenol and standard deviation of triplicates is in parenthesis. Hydrolysis was performed in 1 % (w/V) xylan concentration at 40 °C with a phenol concentration of 0, 10, 100 or 1000 mg/ml.

	TrXyn1			TrXyn2			Xyl40-CD			Xyl40		
Phenol concentration	10 (mg/ml)	100 (mg/ml)	1000 (mg/ml)	10 (mg/ml)	100 (mg/ml)	1000 (mg/ml)	10 (mg/ml)	100 (mg/ml)	1000 (mg/ml)	10 (mg/ml)	100 (mg/ml)	1000 (mg/ml)
acetovanillone	99 (1)	103 (2)	98 (2)	100 (4)	105 (1)	99 (4)	97 (2)	98 (1)	99 (3)	103 (2)	110 (4)	107 (4)
p-coumaric acid	96 (3)	95 (2)	102 (1)	100 (3)	99 (3)	100 (1)	102 (2)	96 (1)	93 (0)	92 (6)	97 (5)	76 (6)
ferulic acid	111 (1)	112 (3)	113 (1)	100 (1)	100 (0)	98 (2)	105 (4)	96 (10)	106 (6)	96 (10)	96 (10)	87 (3)
homovanillyl alcohol	104 (2)	100 (3)	101 (3)	106 (3)	101 (3)	106 (1)	101 (1)	103 (4)	102 (2)	106 (8)	105 (15)	96 (6)
protocatechuic acid	107 (1)	102 (2)	106 (3)	102 (2)	102 (1)	102 (1)	100 (2)	103 (2)	102 (2)	104 (5)	105 (6)	98 (6)
syringaldehyde	98 (5)	87 (2)	50 (4)	102 (1)	100 (2)	94 (3)	101 (1)	100 (4)	91 (5)	105 (8)	102 (4)	83 (11)
syringic acid	101 (1)	109 (2)	103 (5)	97 (1)	102 (3)	101 (1)	93 (5)	98 (2)	95 (3)	104 (3)	109 (4)	96 (4)
vanillic acid	95 (3)	99 (4)	108 (4)	114 (1)	104 (8)	105 (4)	101 (1)	102 (2)	98 (6)	113 (8)	108 (2)	93 (3)
vanillin	85 (4)	90 (5)	102 (9)	101 (1)	102 (1)	102 (3)	104 (2)	102 (1)	101 (3)	109 (11)	110 (6)	122 (2)
